# Effects of exercise intensity on vascular and autonomic components of the baroreflex following glucose ingestion in adolescents

**DOI:** 10.1007/s00421-019-04076-y

**Published:** 2019-02-07

**Authors:** Ricardo S. Oliveira, Alan R. Barker, Florian Debras, Sascha H. Kranen, Craig A. Williams

**Affiliations:** 1 Children’s Health and Exercise Research Centre, Sport and Health Sciences, College of Life and Environmental Sciences, University of Exeter, St Luke’s Campus, Exeter, EX1 2LU UK; 2 Astrophysics Group, University of Exeter, Exeter, EX4 4QL UK; 3 Ecole Normale Supérieure de Lyon, CRAL, UMR CNRS 5574, 69364 Lyon, France

**Keywords:** Youth, Oral glucose tolerance test, Arterial stiffness, Autonomic system

## Abstract

**Purpose:**

To investigate the effects of an oral glucose tolerance test (OGTT) on baroreflex sensitivity (BRS) in a sample of healthy adolescents, and how acute exercise bouts of different intensities alter the effects of the OGTT on BRS.

**Methods:**

Thirteen male adolescents (14.0 ± 0.5 years) completed three conditions on separate days in a counterbalanced order: (1) high-intensity interval exercise (HIIE); (2) moderate-intensity interval exercise (MIIE); and (3) resting control (CON). At ~ 90 min following the conditions, participants performed an OGTT. Supine heart rate and blood pressure were monitored continuously at baseline, 60 min following the conditions, and 60 min following the OGTT. A cross-spectral method (LFgain) was used to determine BRS gain. Arterial compliance (AC) was assessed as the BRS vascular component. LFgain divided by AC (LFgain/AC) was used as the autonomic component.

**Results:**

Although non-significant, LFgain moderately decreased post-OGTT when no exercise was performed (pre-OGTT = 24.4 ± 8.2 ms mmHg^− 1^; post-OGTT = 19.9 ± 5.6 ms mmHg^− 1^; ES = 0.64, *P* > 0.05). This was attributed to the decrease in LFgain/AC (pre-OGTT = 1.19 ± 0.5 ms µm^− 1^; post-OGTT = 0.92 ± 0.24 ms µm^− 1^; ES = 0.69, *P* > 0.05). Compared to CON (Δ = − 4.4 ± 8.7 ms mmHg^− 1^), there were no differences for the pre–post-OGTT delta changes in LF/gain for HIIE (Δ = − 3.5 ± 8.2 ms mmHg^− 1^) and MIIE (Δ = 1.3 ± 9.9 ms mmHg^− 1^) had no effects on BRS following the OGTT (all ES < 0.5). Similarly, compared to CON (Δ = − 0.23 ± 0.40 ms µm^− 1^) there were no differences for the pre–post-OGTT delta changes in LF/gain for HIIE (Δ = − 0.22 ± 0.49 ms µm^− 1^) and MIIE (Δ = 0.13 ± 0.36 ms µm^− 1^).

**Conclusion:**

A moderate non-significant decrease in BRS was observed in adolescents following a glucose challenge with no apparent effects of exercise.

## Introduction

Atherosclerosis has its origins during childhood with elevated blood pressure contributing to plaque formation independently of other cardiovascular disease risk factors in youth (Franks et al. 2010; McGill et al. 2001). A sentinel for hypertension development is decreased baroreflex sensitivity (BRS). In young adults, a lower BRS is present in normotensive children of hypertensive parents (Boutcher et al. 2011), and impaired BRS is associated with high blood pressure in normotensive adolescents (Fitzgibbon et al. 2012; Honzikova and Zavodna 2016). These studies indicate BRS dysfunction may be associated with cardiovascular disease burden in youth and is worthy of further research so as to inform preventative health strategies. Baroreflex sensitivity is composed of autonomic and vascular components which contribute towards the beat-to-beat detection and adjustment of blood pressure fluctuations (Hunt et al. 2001). Using ultrasound (Taylor et al. 2014; Tzeng 2012), the contribution of the autonomic and vascular determinants of BRS can be non-invasively estimated in a reliable manner (Oliveira et al. 2018b), and are ideally suited for studying BRS in paediatric groups.

In non-diabetic children, glucose intolerance assessed during an oral glucose tolerance test (OGTT) predicts adult premature death (Franks et al. 2010). The metabolic effects of elevated blood glucose (GLU) concentration ([GLU]) following an OGTT have implications for the arterial and autonomic systems, as evidenced by decreased autonomic modulation and increased vascular stiffness in diabetic adolescents (Shin et al. 2010), which may contribute to chronic BRS dysfunction in youth with diabetes (Honzikova and Zavodna 2016). However, a lowered BRS caused by a rise in [GLU] is not only observed in diseased populations. For example, a decreased BRS has been reported in healthy adults during an OGTT, which was attributed to a diminished autonomic determinant (Holwerda et al. 2015). The mechanism by which glucose decreases BRS remains controversial, however, as a rise in [GLU] leads to lowered vagal modulation (Cao and Pilowsky 2014; Holwerda et al. 2015; Lefrandt et al. 2000) (i.e., reduced autonomic component), and increased common carotid artery (CCA) stiffness due to endothelial dysfunction (Wilkinson et al. 2004; Zhu et al. 2007) (i.e., reduced vascular component). Although growth and maturation are associated with an augmented BRS due to maturation of the autonomic component (Lenard et al. 2004), the influence of a glucose load on the BRS and its associated mechanisms is unknown in youth. As glucose intolerance is associated with poor vascular and autonomic functions in diabetic youth (Shin et al. 2010), it is plausible that elevated [GLU] may reduce BRS in healthy adolescents. A better understanding of the BRS physiology under different challenges, such as during an OGTT, can help inform strategies to target cardiovascular disease risk reduction in paediatric groups.

Physical activity is an important strategy to improve glucose metabolism (Henderson et al. 2012), and is also positively associated with autonomic and vascular functions in children and adolescents (Fernhall and Agiovlasitis 2008; Oliveira et al. 2017). While in adults temporally decreasing physical activity levels does not exacerbate the deleterious effects of an OGTT on BRS (Holwerda et al. 2015), the possible effect of increasing physical activity via prior exercise on the subsequent BRS responses to an OGTT is currently unknown. In healthy adolescents, a single bout of high- and moderate-intensity exercise has been shown to reduce the increase in blood [GLU] during an OGTT (Cockcroft et al. 2015, 2017b), suggesting that acute exercise may alter the BRS responses to an OGTT by lowering blood [GLU]. Additionally, in the hours following high- but not moderate-intensity exercise, improvements in arterial function are observed in healthy adolescents (Bond et al. 2015), showing that exercise may preserve the vascular component of BRS. As the intensity of exercise has recently been proposed to be a determinant of cardiovascular disease risk reduction in youth (Barker et al. 2018; Carson et al. 2014), elucidating whether exercise of different intensities alters the BRS response to an OGTT will further contribute to our understanding of cardiovascular disease risk reduction in youth.

The aims of the present study were to investigate in healthy adolescents: (1) the effect of an OGTT on BRS and its vascular and autonomic components; and (2) whether an acute bout of moderate- and high-intensity exercise alters the effects of an OGTT on BRS and its associated mechanisms. It was hypothesised that (1) the OGTT would impair BRS via decrease in the autonomic and vascular determinants; (2) a prior bout of moderate- and high-intensity exercise would lead to a significant lower [GLU] concentration from OGTT compared to a non-exercise control situation (CON); and (3) that both high-intensity interval exercise (HIIE) and moderate-intensity interval exercise (MIIE) would maintain BRS at baseline values following the OGTT due to preserved vascular and autonomic components.

## Methods

### Participants

Sample size was calculated a priori using G*Power aiming to detect a moderate effect size (ES) of 0.78 observed by Holwerda et al. (2015) with a test–retest relationship of 0.65 for the LFgain (Oliveira et al. 2018b). With the inclusion of statistical power of 80% and an alpha of 5%, the sample size needed was thirteen. Thirteen healthy male adolescents (14.0 ± 0.5 years; body mass index = 18.6 ± 3.0 m kg^− 2^; body fat = 12.0 ± 4.7%; VO_2_ max = 50.9 ± 5.3 mL kg^− 1^ min^− 1^) volunteered to take part in this investigation. The status of pubertal development, as measured using five stages of pubic hair development (Morris and Udry 1980) was: stage 2 *n* = 3, 3 *n* = 1, 4 *n* = 8, 5 *n* = 1. Before participating in the study, participants and parents completed a health questionnaire and all participants were free of conditions, such as diabetes, hypertension, asthma, or any disease altering autonomic and vascular functions. Assent and consent were obtained from participants and parents/guardians, respectively, and all procedures were approved by the institutional ethics committee (approval number: 160217/B/04).

### Experimental design

Participants completed four visits to the laboratory with a minimum of 72 h between each visit and no more than 4 weeks to finish all visits. The visits were all conducted in the morning following an overnight fast, as detailed below:

*Visit 1* Participants were familiarised to the BRS protocol and treadmill running. Stature and body mass were measured, followed by triceps and subscapular skinfolds taken in triplicate for estimation of body fat percentage using age- and sex-specific validated equations (Slaughter et al. 1988). Participants then completed an incremental test combined with a supramaximal bout to exhaustion (Barker et al. 2011), with breath-by-breath gas exchange measurements (Cortex Metalyzer III B, Germany). The incremental test started at 6 km·h^− 1^ with 1% inclination after a 3-min warm-up at 4 km h^− 1^ (Woodway GmbH, Germany). Increments of 0.5 km h^− 1^ were completed every 30 s until participants reached exhaustion, when maximal aerobic speed was determined. Following 10 min of recovery, participants completed a running bout to exhaustion with 5% inclination at the MAS obtained in the incremental test. Maximum oxygen uptake (VO_2_max) was measured as the highest value obtained in either the ramp or the supramaximal tests. The gas exchange threshold was visually obtained from the incremental test data as a disproportionate increase in carbon dioxide production relative to VO_2_. At the end of this visit, to assure parity between visits, participants received food diaries and accelerometers, which were used in the 48 h preceding visits 2–4.

**Fig. 1:**
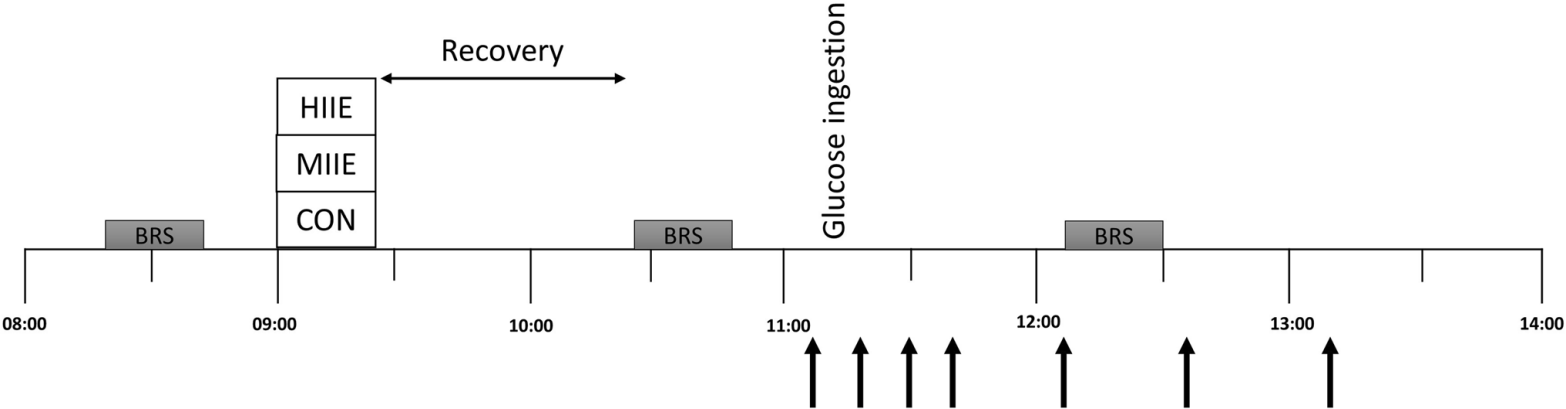
Overall scheme of visits 2–4. Black arrows: blood sampling. *HIIE* High-intensity interval exercise, *MIIE* moderate-intensity interval exercise, *CON* control, *BRS* baroreflex sensitivity protocol

*Visits 2–4* The outline of this visit is presented in the Fig. 1. Before these visits, participants were instructed to avoid strenuous exercise and to keep a similar diet obtained from the food diaries. Following an overnight fast, participants were transported to the laboratory and completed the baseline measurements between 8 and 9 am. A three-lead electrocardiography and a finger cuff (Finometer PRO, Netherlands) were fitted and the BRS protocol started after 10 min of supine rest in a temperature- (21–24 °C) and light-controlled room. The BRS protocol consisted of: (1) a measurement of brachial blood pressure to calibrate the Finometer for brachial reconstructed blood pressure assessment (Guelen et al. 2008); this device has been validated to monitor blood pressure in children (Tanaka et al. 1994); (2) after calibration, images of the CCA were recorded for 15 cardiac cycles; and (3) participants were instructed to pace their breathing frequency at 12 cycles per min for 5 minutes (Tzeng 2012; Tzeng et al. 2009; Williams and Lopes 2002). The procedures were completed in the described order and lasted ~ 20 min (including the 10 min rest preceding the protocol).

Following the baseline assessments, participants completed in a counterbalanced order (i.e., a similar distribution between conditions at visits 2–4), the following experimental conditions on separated days: (1) HIIE; (2) MIIE; and (3) CON. HIIE consisted of eight 1-min bouts at 90% of the maximal aerobic speed, interspersed by 75 s of walking at 4 km h^− 1^. MIIE consisted of 1-min bouts at 90% of gas exchange threshold, interspersed by 75 s of walking at 4 km h^− 1^. The number of bouts during MIIE was calculated on an individual basis to match the total distance covered in the HIIE protocol. Both HIIE and MIIE were preceded by a 3-min warm-up and a 2-min cool down at 4 km h^− 1^. For CON, participants pursued sedentary activities in a seated position, such as computer and board games.

At 60 min following the experimental conditions (pre-OGTT), participants repeated the BRS protocol. Following this BRS measurement, an OGTT took place (~ 90 min post-completion of the experimental conditions). For this, participants consumed 75 g of glucose dissolved in 300 mL of water. Fingertip blood samples were collected at 0, 10, 20, 30, 60, 90 and 120 min for assessment of plasma [GLU] (YSI, 2300 Stat Plus Glucose analyser, Yellow Springs, USA), as previously described (Cockcroft et al. 2015). The OGTT has been shown to be reliable in a sample of healthy adolescents (i.e., observed test–retest coefficient of variation of 5–7% for glucose-derived indices [Cockcroft et al. 2017a)]. Participants repeated the BRS protocol 60 min post-OGTT (post-OGTT), as it has been shown that BRS is reduced at this time point following an OGTT in adults (Holwerda et al. 2015).

### Baroreflex sensitivity analysis

**Fig. 2:**
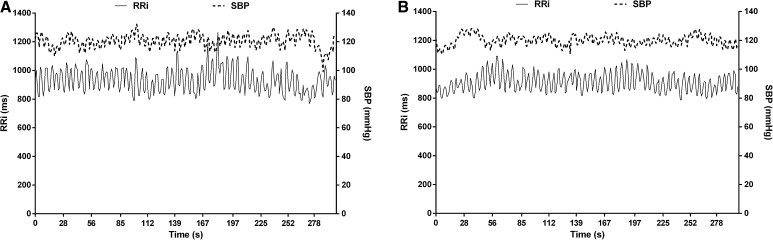
Systolic blood pressure and RR interval time series of a representative participant pre- and post-OGTT

**Fig. 3:**
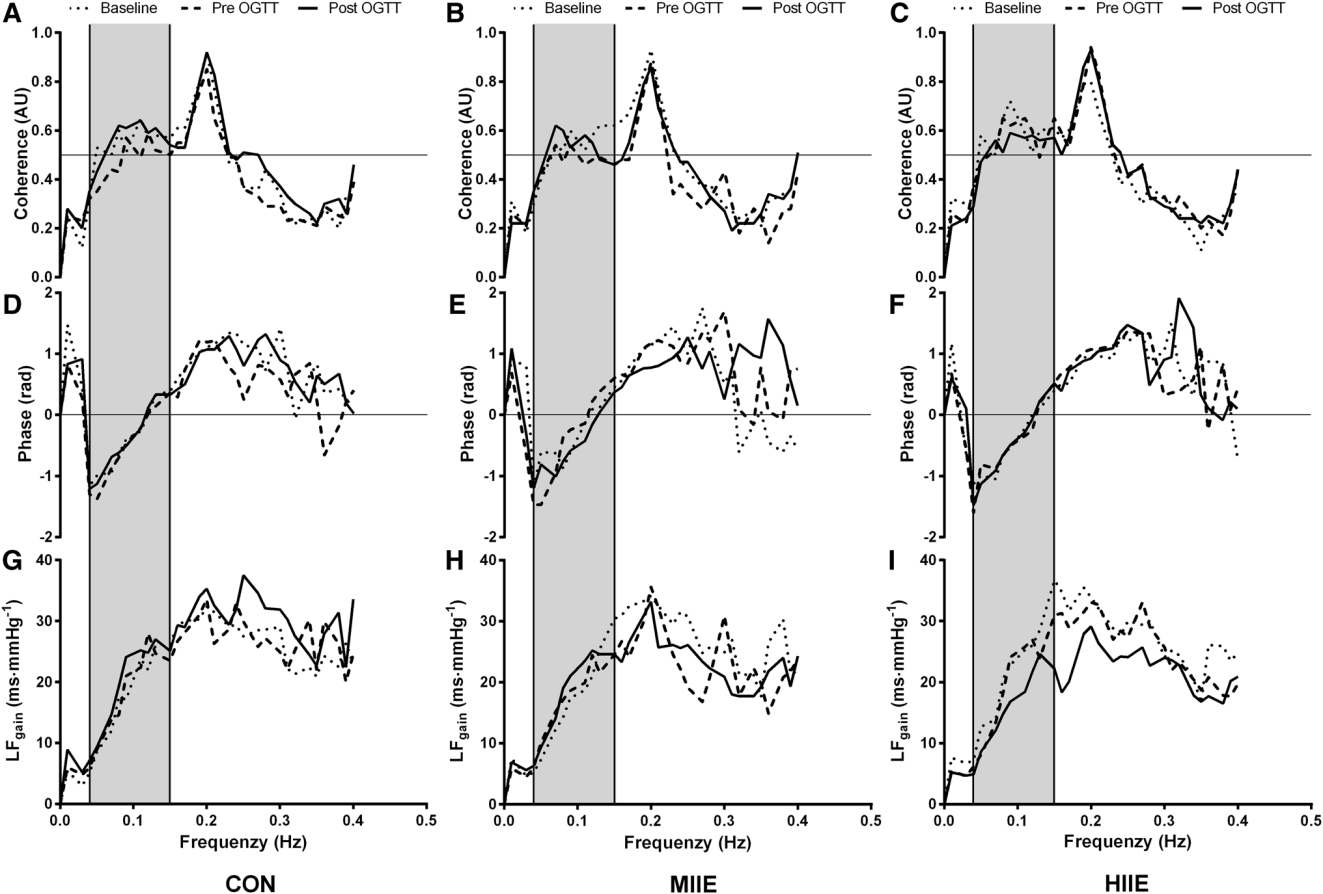
Averaged (*n* = 12) phase, coherence and LFgain at baseline, pre- and post-OGTT for CON, HIIE and MIIE. Shadowed area represents the region of interest (i.e., low frequency range)

BRS analysis procedures in the present study were performed according to the previous studies using validated methods (Chirico et al. 2015; Lenard et al. 2004; Robbe et al. 1987; Saul et al. 1991). Electrocardiography and blood pressure were recorded simultaneously at 1000 Hz (PowerLab, ADInstruments). RR intervals and systolic blood pressure data were extracted and saved for later analysis. Ectopic beats were automatically identified and linear interpolation with a low filter was applied when < 3% error was present (Kubios v3.0) (Tarvainen et al. 2014). Systolic blood pressure trace was visually checked, and errors manually replaced by linear interpolation using adjacent systolic blood pressure. Systolic blood pressure and RR intervals were visually checked before data analysis and a representative sample is presented in Fig. 2. Integrated gain (LFgain) of BRS was determined from the final 5 minutes of the BRS protocol. For this purpose, beat-to-beat RR interval and brachial reconstructed systolic blood pressure were interpolated at 2 Hz, de-trended using a linear function and filtered using a Butterworth filter set to 0.95 Hz. A fast Fourier transformation was then applied using the Welch method to obtain the power spectrum in the low frequency (LF = 0.04–0.15 Hz) band and a cross-spectral transfer function was then calculated (LFgain) to evaluate baroreflex gain, defined as the average of the cross-spectrum divided by the power spectrum of systolic blood pressure in the range where the coherence was > 0.5, hence expressed in ms mmHg^−1^. This index was chosen due to its established validity compared to BRS assessment using vasoactive drugs (Di Rienzo et al. 2001; Persson et al. 2001). In addition to the averaged values described in the tables and figures, we present in Fig. 3 the mean values of phase, coherence and LFgain for all three conditions and participants over the frequency spectrum of interest. To highlight the feedback nature of BRS, the negative phase can be noted over the LF range, indicating that low frequency of systolic blood pressure peaked before the observed low frequency of RR intervals. On the contrary, the HF range does not reflect baroreflex mechanisms but rather respiratory sinus arrhythmia, as evidenced in the peak coherence observed at 2.0 Hz (i.e., breathing frequency).

### Vascular and autonomic determinants

All CCA images were recorded ~ 2 cm distal from the carotid bulb using a high-resolution (~ 13 MHz) linear array transducer (Apogee, 1000, SIUI, China). The images were obtained over 15 cardiac cycles recorded at 15 frames per second. Subsequently, CCA images were analysed using validated wall tracking software (Carotid Analyzer, Medical Imaging Applications LLC) (Mancini et al. 2004) for determination of diastolic lumen diameter and systolic lumen diameter. The average of 3–7 cardiac cycles with clear definitions of the near and far walls was used. During the 15 cardiac cycles, beat-to-beat brachial reconstructed blood pressure (Guelen et al. 2008) was averaged and used to determine pulse pressure. The vascular components of BRS were determined according to previously published literature as follows (Laurent et al. 2006):

$${\text{Arterial compliance}}-{\text{AC }}\left( {\upmu {\text{m}} \times {\text{mmH}}{{\text{g}}^{ - {\text{1}}}}} \right)=\Delta {\text{D}}/{\text{PP,}}$$

where ΔD is systolic lumen diameter minus diastolic lumen diameter, and PP is the obtained pulse pressure;

$${\text{Arterial distensibility }}-{\text{AD }} ({\text{mmH}}{{\text{g}}^{ - {\text{1}}}} \times {\text{1}}{0^{ - {\text{3}}}})=\Delta {\text{CSA}}/{\text{PP}}\cdot{\text{CSAmin}},$$

where CSA in the cross-sectional CCA artery calculated as CSA = *πr*^2^ being *r* = diameter/2 and ΔCSA the maximal CSA minus minimal CSA (CSAmin).

The autonomic and vascular determinants of BRS were determined according to previous study (Lenard et al. 2004). Briefly, AC was considered as the vascular component of the BRS and expressed as µm mmHg^− 1^. To calculate the autonomic determinant, LFgain was divided by the AC and expressed as LFgain/AC in ms µm^− 1^.

### Food and physical activity standardisation

Participants were asked to avoid vigorous exercise in the 48 h preceding the experimental visits. Accelerometers (GENEActiv, Activinsigths Ltd, UK) were used to estimate the amount of moderate-to-vigorous physical activity (MVPA) in the 48 h preceding the experimental visits. For this, data were collected at 100 Hz and treated using freely available spreadsheets (http://www.geneactive.org). MVPA was obtained using population specific cutoffs (Phillips et al. 2013). Participants were also asked to keep a similar diet in the 48 h before the experimental visits to assure parity between the visits. Food diaries were used to compare total calories and the percentage contribution from carbohydrates, lipids and proteins (CompEat Pro, UK).

### Statistical analysis

Data are presented as means and standard deviation. Physiological responses to exercise were investigated using paired *t* test. To test the first aim of this study, the effects of the OGTT on the physiological parameters, paired *t* test was performed from CON group pre- and post-OGTT. To test possible differences between outcomes at baseline, repeated-measures ANOVA with three levels for condition was applied. As no differences were found for any variables at baseline, to test aim 2 of this study, delta changes pre- and post-OGTT were calculated for each condition (HIIE, MIIE and CON) and the differences between conditions were tested using one-way repeated-measures ANOVA. Total area under the curve (TAUC) and incremental area under the curve (IAUC) analyses quantified the plasma [GLU] responses to the OGTT using the trapezium rule (GraphPad Prism 6.02, USA) and differences between conditions were tested using repeated-measures ANOVA. Sphericity was tested using Mauchly’s test and when violated corrections were performed using Greenhouse-Geisser. Post hoc comparisons were applied when adequate using the least square difference procedure. Analyses were performed using SPPS v.22, with the alpha level set at 0.05. Finally, the magnitude of mean differences was interpreted using ES: ≥0.2 small, ≥ 0.5 moderate, ≥ 0.8 large (Cohen 1977).

## Results

One participant was excluded from the BRS assessment due to errors in the electrocardiographic signal, and two from the CCA analysis due to technical issues with the ultrasound. For clarity, the final sample size for each analysis is described in the Figures and Tables. In the 48 h before the experimental visits, there were no significant differences in the amount of MVPA (HIIE = 117 ± 49, MIIE = 117 ± 32, CON = 111 ± 45 min day^− 1^; *P* = 0.91), energy intake (HIIE = 1987 ± 732, MIIE = 1912 ± 458, CON = 2079 ± 643 kcal day^− 1^; *P* = 0.55) and relative macronutrient contribution (carbohydrates: HIIR = 52 ± 7, MIIE = 50 ± 5, CON = 51 ± 8%; lipids: HIIE = 32 ± 1, MIIE = 32 ± 1, CON = 33 ± 1%; proteins: HIIE = 16 ± 3, MIIE = 17 ± 3, CON = 16 ± 4%; all *P* > 0.05) between the experimental conditions. All effect sizes between conditions were considered small (all ES < 0.5).

HIIE elicited significantly greater peak VO_2_ [%of max] (2.2 ± 0.2 [89%] vs 1.6 ± 0.1 [66%] L min^− 1^; *P* < 0.001, ES = 3.8), and average HR [%of max] [154 ± 3 (78%) vs 128 ± 5 (64%) beats-per-minute; *P* < 0.001, ES = 6.3] compared to MIIE. HIIE was significantly shorter in duration than MIIE (21.8 ± 0 vs 28.0 ± 1.8 min; *P* < 0.001) and the range of bouts in MIIE was 9–12 vs 8 in the HIIE condition.

### OGTT

**Fig. 4:**
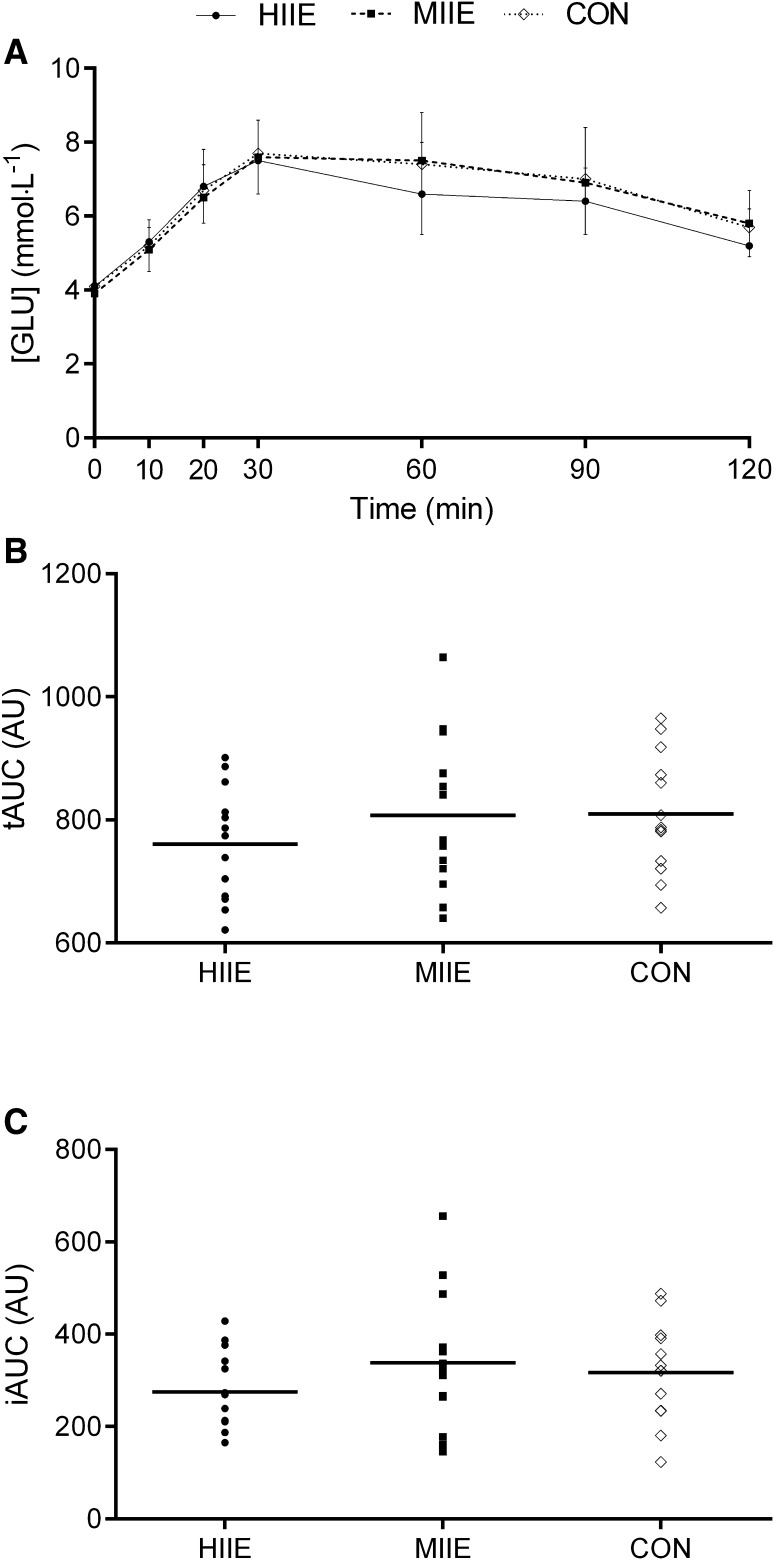
Effects of the experimental condition on: **a** [GLU] (*n* = 13), **b** total area under the curve (*n* = 13) for the different conditions; and **c** incremental area under the curve (*n* = 13)

OGTT responses are depicted in Fig. 4. As expected, the OGTT resulted in an increase in [GLU] over time (*P* < 0.001), but no condition by time interaction (*P* = 0.11) was observed. There was no condition main effect for the TAUC (*P* = 0.12) and IAUC (*P* = 0.15) analysis of the [GLU] response to the OGTT. However, a moderate reduction in IAUC (ES = 0.51) and TAUC (ES = 0.52) for [GLU] was observed for HIIE vs MIIE and HIIE vs CON, respectively.

### Baroreflex sensitivity

The baseline values of LFgain, AC and LFgain/AC obtained between 8 and 9 am for all conditions were: LFgain: HIIE = 24.4 ± 5.8, MIIE = 20.9 ± 4.9, CON = 21.7 ± 5.6 ms mmHg^−1^; AC: HIIE = 19.1 ± 4.1, MIIE = 19.2 ± 3.5, CON = 20.4 ± 3.4 µm mmHg^− 1^; and LFgain/AC: HIIE = 1.32 ± 0.5, MIIE = 1.07 ± 0.4, CON = 1.14 ± 0.3 ms µm^− 1^.

Pre- and post-OGTT LFgain (pre = 24.4 ± 8.2, post-OGTT = 19.9 ± 5.6 ms mmHg^−1^; *P* = 0.11; ES=-0.63), AC (pre = 20.4 ± 4.2, post-OGTT = 22.6 ± 5.8 µm mmHg^− 1^; *P* = 0.22; ES = 0.55), and LFgain/AC (pre = 1.19 ± 0.5, post-OGTT = 0.92 ± 0.2 ms µm^− 1^; *P* = 0.07; ES = −0.63) were not significantly altered for CON, although moderate effects were observed for all comparisons.

**Fig. 5:**
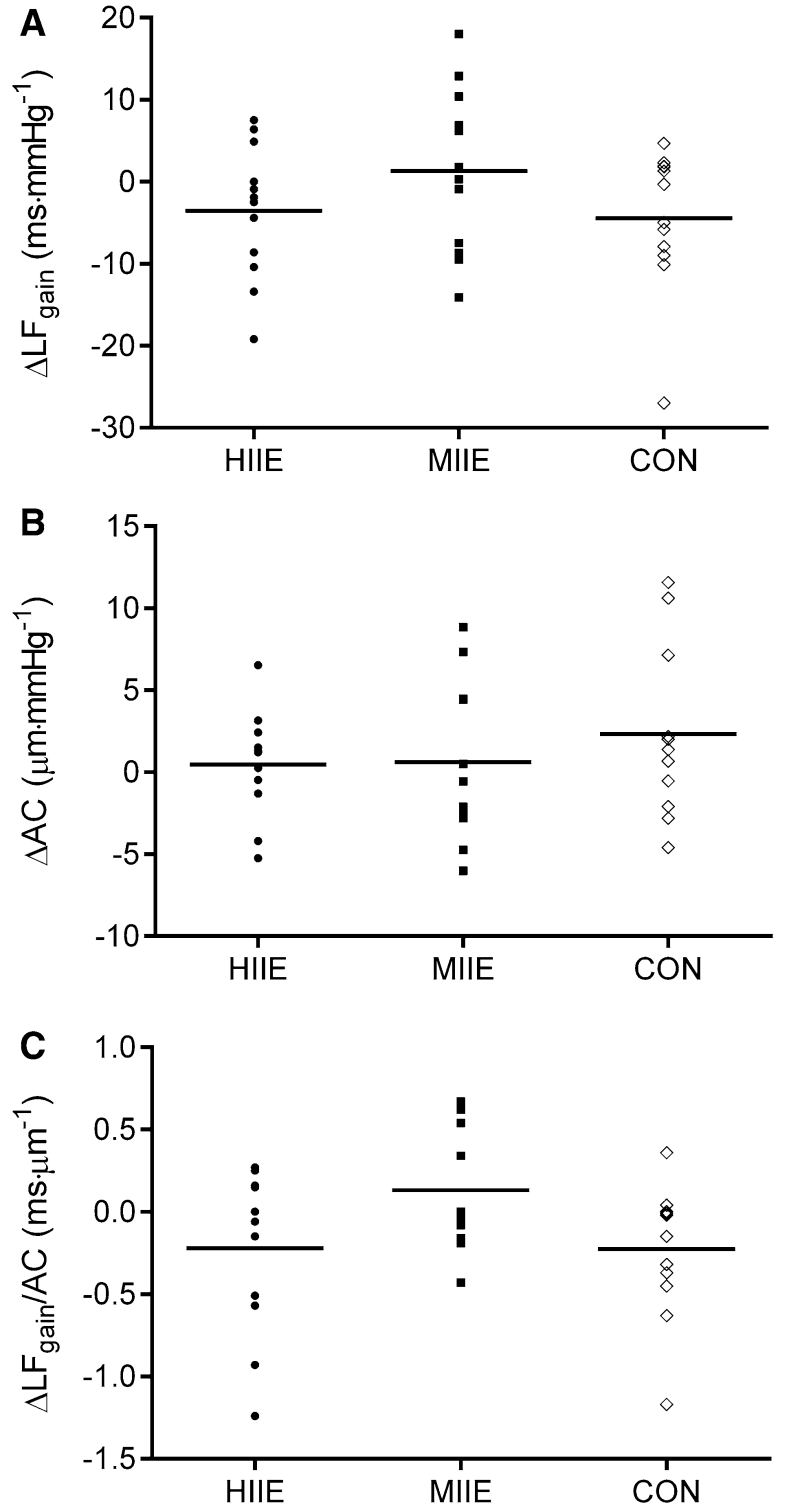
Effects of the experimental condition on the delta changes pre- and post-OGTT for **a** LFgain (*n* = 12); **b** AC (*n* = 11); and **c** LFgain/AC (*n* = 10)

To test the effects of conditions, delta changes were calculated for HIIE (pre- and post-OGTT for LFgain = 22.5 ± 9.6 and 18.9 ± 8.5 ms mmHg^−1^; *P* = 0.16; ES = − 0.39; AC = 19.8 ± 3.1 and 20.3 ± 2.8 µm mmHg^− 1^*P* = 0.41; ES = 0.16; and LFgain/AC = 1.16 ± 0.6 and 0.90 ± 0.4 ms µm^− 1^*P* = 0.13; ES = 0.50, respectively) and MIIE (pre- and post-OGTT for LFgain = 21.4 ± 7.2 and 22.8 ± 9.1 ms mmHg^−1^*P* = 0.68; ES = 0.16; AC = 21.1 ± 4.0 and 21.8 ± 4.4 µm mmHg^− 1^*P* = 0.98; ES = 0.15; and LFgain/AC = 0.94 ± 0.3 and 1.10 ± 0.5 ms µm^− 1^*P* = 0.13; ES = 0.38, respectively). Comparing delta changes between conditions, no effects were observed for LFgain (*P* = 0.31), AC (*P* = 0.63), and LFgain/AC (*P* = 0.10) pre- and post-OGTT (Fig. 5). All effect sizes between conditions were considered small (all ES < 0.5).

### Common carotid artery

CCA properties are presented in Table 1. Diastolic lumen diameter (*P* = 0.004; ES = − 0.21) and ΔD (*P* = 0.048; ES = 0.30) were significantly altered by the OGTT during CON. No significant effects were observed for systolic lumen diameter pre and post-OGTT (*P* = 0.30; ES = 0.09). When the pre–post-OGTT delta changes were compared between CON, HIIE and MIIE, no effect of condition was observed for diastolic lumen diameter (*P* = 0.12), systolic lumen diameter (*P* = 0.51), and ΔD (*P* = 0.40). All effect sizes between conditions were considered small (all ES < 0.5).

**Table 1:** Common carotid, blood pressure and heart rate variability outcomes

	HIIE			MIIE			CON		
	Pre-OGTT	Post-OGTT	Δ	Pre-OGTT	Post-OGTT	Δ	Pre-OGTT	Post-OGTT	Δ
DLD (µm) *n* = 11	5425.5 ± 324.7	5465.5 ± 284.0	40.0 ± 162.4	5417.3 ± 309.2	5377.3 ± 387.5	− 40.0 ± 148.5	5400.0 ± 364.4	5323.6 ± 370.5	− 76.4 ± 67.0
SLD (µm) *n* = 11	6305.5 ± 344.1	6335.5 ± 229.9	30.0 ± 207.4	6322.7 ± 304.3	6300.0 ± 341.6	− 22.7 ± 118.1	6268.2 ± 387.6	6234.5 ± 355.0	− 33.6 ± 103.0
ΔD (µm) *n* = 11	880.0 ± 144.9	870.0 ± 131.8	− 10 ± 106.4	905.5 ± 116.2	922.7 ± 139.2	17.3 ± 122.1	868.2 ± 143.2	910.9 ± 137.7	42.7 ± 62.8
SBP (mmHg) *n* = 13	108 ± 10	110 ± 13	1.5 ± 9.8	113 ± 9	114 ± 15	0.3 ± 10.7	111 ± 11	110 ± 12	− 0.6 ± 9.4
DBP (mmHg) *n* = 13	61 ± 6	65 ± 9	3.9 ± 6.8	69 ± 7	67 ± 11	− 1.4 ± 9.6	65 ± 6.7	66 ± 7	0.82 ± 6.4
MAP (mmHg) *n* = 13	75 ± 6*	78 ± 10	3.2 ± 7.0	82 ± 7	81 ± 11	− 0.9 ± 8.7	78.9 ± 6.5	79 ± 7	0.14 ± 5.9
HFln (ms^2^) *n* = 12	8.5 ± 0.9	8.3 ± 0.8	− 0.23 ± 0.73	8.5 ± 1.0	8.4 ± 0.9	− 0.06 ± 0.79	8.4 ± 0.7	8.8 ± 0.7	0.39 ± 0.43
LFln (ms^2^) *n* = 12	7.3 ± 1.2	7.0 ± 1.0	− 0.29 ± 0.61	7.2 ± 0.8	7.3 ± 0.7	0.11 ± 0.60	7.5 ± 0.6	7.4 ± 0.6	− 0.15 ± 0.60

*HIIE* High-intensity interval exercise, *MIIE* moderate-intensity interval exercise, *CON* control, *DLD* diastolic lumen diameter, *SLD* systolic lumen diameter, *SBP* systolic blood pressure, *DBP* diastolic blood pressure, *MAP* mean arterial pressure, *HF* high frequency, *LF* low frequency

*P < 0.05 compared to MIIE. For *P* values and effect sizes refer to text

### Blood pressure and HRV

Blood pressure and HRV are presented in Table 1. Systolic blood pressure (*P* = 0.11; ES = − 0.63), diastolic blood pressure (*P* = 0.22; ES = 0.55), mean arterial pressure (*P* = 0.07; ES = − 0.63), and LF (*P* = 0.07; ES = − 0.63) were not significantly altered by the OGTT but exhibited moderate effects. On the contrary, post-OGTT high frequency significantly increased moderately (*P* = 0.010; ES = 0.54). When the pre–post-OGTT delta changes were compared between CON, HIIE and MIIE, there was no effect of condition for the delta changes in systolic blood pressure (*P* = 0.85), diastolic blood pressure (*P* = 0.28), mean arterial pressure (*P* = 0.36). Similarly, no effects of condition were observed for the delta changes in low frequency (*P* = 0.63) and high frequency (*P* = 0.10) pre- and post-OGTT. All effect sizes between conditions were considered small (all ES < 0.5).

## Discussion

To our knowledge, this is the first study to examine the influence of hyperglycaemia, delivered using an OGTT, on BRS in health adolescents. The OGTT caused a moderate but non-significant decrease in BRS and its autonomic determinant. Another novel feature of the current study is that we examined the role of different exercise intensities performed ~ 90 min prior to the OGTT on the changes in BRS, [GLU], and hemodynamics. The main findings regarding exercise were: (1) although non-significant, HIIE but not MIIE moderately decreased the glucose responses to an OGTT; and (2) exercise performed 90 min prior the OGTT had no effect on BRS following the OGTT.

### Effects of glucose on BRS

This is the first study in a sample of healthy adolescents investigating the impact of an acute glucose load on the mechanistic control of blood pressure. To date, most researches investigating BRS during metabolic challenges have been performed in adults, or in populations with diabetes, obesity, or elevated blood pressure (Holwerda et al. 2015; Malin et al. 2016b; Straznicky et al. 2009). In the present study, the increase in blood [GLU] following the OGTT led to a moderate (i.e., ES = -0.64) yet non-significant decrease in the LFgain in the CON condition. Although our findings failed to reject the null hypothesis, the magnitude of the observed changes is similar to the study by Holwerda et al. (Holwerda et al. 2015) who reported a moderate and significant decrease (pre-OGTT = 20 ± 9; post-OGTT = 14 ± 6; ES = 0.78) in LFgain 60 min following the ingestion of a glucose load in healthy adults. The decrease in BRS appears to be moderated by increase in [GLU], because BRS at 60 min post a hyperinsulinemic euglycemic clamp did not decrease compared to baseline, suggesting that glucose is responsible for a decreased BRS when [GLU] peaks at around 7.5 mmol L^− 1^ (Holwerda et al. 2015). We further extend the findings of Holwerda et al. (2015) by investigating the likely mechanisms by which [GLU] leads to decrease in BRS by estimating the autonomic and the vascular determinants of BRS. Our present observations demonstrated that although non-significant, a moderate effect (ES = 0.63) was observed for the OGTT on the changes in the autonomic marker of BRS, measured as the LFgain/AC. These results are in accordance with adult data showing a lowered autonomic modulation caused by a rise in blood [GLU] (Cao et al. 2016; Cao and Pilowsky 2014), and may provide a mechanism linking cross-sectional findings of a lowered vagal modulation and impaired BRS in children with diabetes (Honzikova and Zavodna 2016).

We also investigated the effects of [GLU] on the vascular determinant of BRS measured as CCA compliance (Lenard et al. 2004). A decrease in CCA compliance, therefore, would be an indicative of a decreased vascular determinant of the BRS (Oliveira et al. 2018b), and consequently a decrease in the overall BRS. As arterial compliance is partially dependent on endothelial function (Wilkinson et al. 2004), the lack of decrease in CCA compliance in the present study may be explained by previous literature showing no decrease in endothelial function in normal weight children during an OGTT (Dengel et al. 2007). Moreover, and different to the present study, adult studies have shown increase in arterial stiffness, assessed as pulse wave velocity, following an OGTT (Baynard et al. 2009; Kobayashi et al. 2018). Differences in the arterial stiffness assessment method (i.e., CCA compliance and distensibility vs central and peripheral pulse wave velocity), or differences in arterial stiffness due to ageing (Lenard et al. 2004), may explain discrepancies between the present study and the adult literature. Alternatively, it is possible that the lack of [GLU] effects on CCA compliance in the present study reflects the aerobic fitness of the participants. For example, Kobayashi et al. (Kobayashi et al. 2015), provided data showing increase in central arterial stiffness of participants with lower (38.8 ± 1.9 mL kg^− 1^ min^− 1^), but not with higher VO_2_max (50.2 ± 2.7 mL kg^− 1^ min^− 1^) following a glucose challenge. The present sample had a VO_2_max of 50.9 ± 5.3 mL kg^− 1^ min^− 1^, which may have conferred protection against an increase in arterial stiffness due to hyperglycaemia. Unfortunately, the homogenous nature of the present sample for VO_2_max distribution does not allow this hypothesis to be further investigated. Future studies are needed to test the effects of aerobic fitness on vascular stiffness during OGTT in youth.

### Effects of exercise intensity on glucose

In the present investigation, a moderate yet non-significant effect was observed for the reduction in the iAUC and tAUC for [GLU] following HIIE compared to CON and MIIE, respectively (Fig. 3). These results are different to recent investigations, where moderate to large effects were observed for the reduction in iAUC and tAUC for [GLU] following cycling HIIE and continuous moderate exercise in healthy adolescents (Cockcroft et al. 2015, 2017b). A direct comparison between the studies is complex due to the different modes of exercise (i.e., interval running vs interval and continuous cycling), and participants’ maturity characteristics. Similarly, the time between the exercise and the OGTT challenge was different between the investigations, which may influence the findings. In the present investigation, participants undertook ~ 90 min of recovery between the end of the exercise and the ingestion of the glucose load, whereas in Cockcroft et al. (2015), participants ingested the glucose 10 min following exercise. The longer recovery period in the present study (i.e., 90 min) may favour glucose appearance in the blood due to restoration of blood flow to the splanchnic circulation, contrary to a shorter (i.e., 10 min) recovery period, when blood flow is still directed to the muscle and skin and exogenous glucose appearance is lower. Alternatively, because in the 60 min following the exercise, there is an increase in peripheral insulin sensitivity but not hepatic (Malin et al. 2016a), the 90-min recovery in the present study may have facilitated endogenous glucose uptake by the muscle, and when the OGTT started the exogenous source was cleared slowly by the liver and muscle. Perhaps a longer follow-up after the exercise conditions (i.e., 24 h), would provide experimental data on the clearance of blood [GLU], due to a slow phase of glycogen repletion (Cockcroft et al. 2017b). Although insulin was not measured, it is likely that the exercise bouts increased insulin sensitivity, as recently reported in healthy adolescents (Cockcroft et al. 2015).

### Effects of exercise intensity on BRS determinants

This is the first study to investigate the impact of different exercise intensities on BRS following the ingestion of a glucose load, which limits direct comparisons with previous studies. We reasoned that performing exercise before the ingestion of a glucose load would confer vascular protection by blunting possible increase in vascular stiffness following the OGTT, as recently reported in adults (Kobayashi et al. 2018). Our results do not support this hypothesis as no differences were observed between conditions (HIIE, MIIE and CON) for the delta changes in arterial compliance. Recently, the OGTT has been shown to decrease femoral arterial compliance in adults only, when participants’ physical activity levels are decreased for five consecutive days (Credeur et al. 2018), suggesting a protective role of physical activity levels on arterial compliance. As such, although participants refrained from exercise in the 48 h preceding data collection in our present study, the amount of physical activity performed in the week protected against the decrease in vascular compliance following the OGTT. Future studies should test the effects of decrease or increase in physical activity on the autonomic and vascular determinants of BRS in adolescents.

We also reasoned that a decrease in [GLU] following HIIE and MIIE would maintain a BRS value similar to pre-OGTT ingestion. This hypothesis has foundations on the mechanistical link between rise in [GLU] and decrease in BRS recently described in adults (Holwerda et al. 2015). However, the moderate decrease in glucose TAUC and IAUC caused by HIIE did not translate in augmented or maintained BRS and its autonomic determinant as evidenced by the lack of differences between CON and HIIE on the delta changes of LFgain and LFgain/AC. It can be speculated that a higher decrease on [GLU] than the observed in our present study is necessary to keep BRS preserved from the [GLU] effects. No studies exist providing dose–response between GLU and autonomic function.

As with all studies, there are a few limitations that must be recognised. First, although the present method to measure the autonomic determinant of BRS is reliable (Oliveira et al. 2018b), it has not been validated against drug blockade methods. However, our present results and the literature showing the effects of blood [GLU] on autonomic modulation, reinforces the validity of the present measures (Cao et al. 2016; Cao and Pilowsky 2014). Moreover, in youth it is unfeasible and unethical to use drug infusion to test the autonomic determinant as described by Taylor et al. (2014) and an alternative is to use overall BRS gain obtained from the spontaneous index together with arterial compliance (Tzeng 2012). In this scenario, if arterial compliance decreases and overall BRS gain increases, a ratio between these two measures can be indicative of the autonomic determinant as observed previously in youth with ageing (Lenard et al. 2004) and following exercise (Oliveira et al. 2018a). Second, pulse pressure was not measured at the carotid site when measuring CCA distensibility (Steinback et al. 2005). However, our results are comparable to an adolescent investigation measuring pulse pressure at the CCA site (Lenard et al. 2004). We also did not measure insulin and more mechanistic interpretation about the effects of insulin sensitivity is speculative. Finally, we recognise that the present study failed to find statistically significant results due to the small sample size. We aimed to complete the protocol with 13 healthy participants based on adult findings; however, calculating youth sample sizes based on adult studies has limitations due to changes in arterial compliance with ageing (Lenard et al. 2004), and the homogeneous characteristics of the present sample (i.e., similar VO_2_max) may further contribute to the lack of statistical power. For example, *P* value between conditions for delta change in LFgain/AC was 0.10, this indicates that it is possible that we have missed a statistically significant effect of exercise due to type 2 error. Therefore, careful interpretation should be taken when reading the present effects of exercise. Future studies are needed to replicate our observations in healthy youth including a larger sample size and a more heterogeneous group of participants.

## Conclusions

In healthy adolescents, increase in blood [GLU] caused a moderate decrease in BRS likely via decrease in the autonomic BRS determinant. However, the observed effects were non-significant. HIIE and MIIE performed before the ingestion of the glucose load did not have an effect on the observed decrease in BRS. This study provides insights of mechanisms by which exercise could influence BRS responses to blood [GLU].

## Abbreviations

AC: Arterial compliance
ANOVA: Analysis of variance
BRS: Baroreflex sensitivity
CCA: Common carotid artery
CON: Control
GLU: Glucose
HIIE: High-intensity interval exercise
IAUC: Incremental area under the curve
MIIE: Moderate-intensity interval exercise
MVPA: Moderate-to-vigorous physical activity
OGTT: Oral glucose tolerance test
TAUC: Total area under the curve
